# Beyond mindfulness: how Buddhist meditation transforms consciousness through distinct psychological pathways

**DOI:** 10.3389/fpsyg.2025.1649564

**Published:** 2025-08-04

**Authors:** Cheng Wang

**Affiliations:** Research Institute for Ancient Books, Zhejiang University, Hangzhou, China

**Keywords:** Buddhist meditation, consciousness transformation, psychological mechanisms, metacognition, therapeutic efficacy, cultural sensitivity

## Abstract

Buddhist meditation, encompassing practices such as Samatha (focused attention), Vipassana (open monitoring), and Metta (loving-kindness), offers unique pathways for transforming consciousness beyond conventional mindfulness. In this article, we review the studies that explore how these distinct meditative techniques systematically cultivate meta-cognitive insight, emotional regulation, and self-inquiry, facilitating profound shifts in awareness and personal growth. Recent neuroscience and psychology studies show that these techniques influence the mind in different ways: they strengthen attentional stability, reshape self-referential thinking, and reorganize emotional patterns. Such modifications are evident in reorganized brain networks (for example, the default-mode network) and in characteristic EEG patterns. While sharing some parallels with Western mindfulness and hypnosis, Buddhist meditation uniquely emphasizes ethical integration and profound introspection. Challenges remain in objectively measuring advanced meditative states, particularly the experience of “no-self” (anattā), due to the reliance on subjective self-report. Future research should incorporate culturally sensitive methodologies, objective behavioral tasks, and interdisciplinary approaches like neurophenomenology to integrate traditional contemplative wisdom with rigorous scientific inquiry.

## 1 Introduction

Consciousness deepening involves a progression from basic awareness to increasingly refined states of meta-cognitive insight (Travis and Shear, 2010; Poletti et al., 2024). This transformation involves not only noticing thoughts, emotions, and sensory experiences but also adopting a broader perspective on these mental processes. As such, it goes beyond ordinary self-monitoring by altering how one relates to internal phenomena. Recently, neuroscientists have turned their attention to these advanced contemplative states, especially within the context of Buddhist meditation.

Buddhist meditation offers a special way to examine how our minds change. It mainly involves two practices: Samatha (focused attention) and Vipassana (open monitoring). While many Western mindfulness approaches emphasize present-moment awareness, Samatha-Vipassana frameworks integrate both concentrated stabilization and discernment of mental patterns (Sharf, 2015). Consequently, Buddhist meditation presents an opportunity to investigate pathways of consciousness deepening that go beyond symptom reduction or stress management, identifying underlying processes of self-transformation and insight.

Current research is centered on several important areas. First, researchers examine the neurological mechanisms during Buddhist meditation, such as significant shifts in the default mode network (Trousselard et al., 2014; Chowdhury et al., 2023; Laukkonen et al., 2023). Second, work on second-generation mindfulness-based interventions (SG-MBIs) has expanded beyond superficial applications toward deeper clinical integration (Crane et al., 2017; Van Gordon and Shonin, 2020). Finally, debates concerning cultural adaptation and the risks of secular oversimplification raise essential ethical and philosophical questions (Kelly, 2023; Neves-Pereira et al., 2018).

**FIGURE 1:**
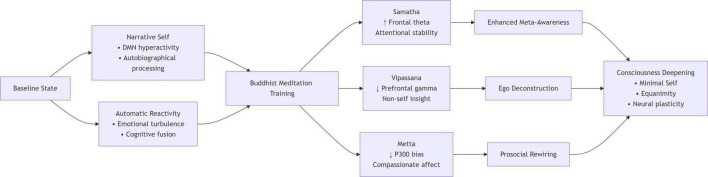
Neurocognitive pathways of consciousness transformation.

Accordingly, this paper aims to (1) explore how Buddhist meditation can foster more profound levels of consciousness, (2) synthesize major psychological findings, and (3) propose directions for future study, including neuroethical considerations and culturally sensitive frameworks. By doing so, it aspires to bridge traditional Buddhist insights and contemporary scientific understandings of mental health and well-being. Figure 1 presents a neurocognitive pathway model of how core Buddhist meditation practices facilitate consciousness transformation in a systematic manner.

## 2 Exploring consciousness: psychological mechanisms of Buddhist meditation

Buddhist meditation traditions transform the mind to a greater depth than what ordinary mindfulness exercises can achieve. By training in a structured way, practitioners learn to notice their thoughts and feelings. This awareness paves the way for insights into the fluid and interdependent nature of experiences. This section explores the conceptual underpinnings guiding these transformations and how they integrate with psychological mechanisms for profound personal change over time.

### 2.1 Buddhist meditation classifications and pathways for deepening consciousness

Buddhist meditation practices focus on organized ways to stabilize and sharpen the mind. This training facilitates the progression of the deepening of consciousness among practitioners. Three core practices—Samatha, Vipassana and Metta—are highlighted both in classical Buddhist scriptures and in modern empirical research. Each of these practices employs distinct mechanisms to transform cognitive processes and emotional states (Lutz et al., 2008; Vago and Silbersweig, 2012; Lippelt et al., 2014; Yordanova et al., 2020).

Samatha, or “focused attention” meditation, involves sustained concentration on a chosen object—often the breath or a visual focus. It aims to achieve a state of absorption. During advanced stages, one may notice a marked decline in distractibility accompanied by greater inner calm (Neri et al., 2024). Concentrative meditation (e.g., Samatha) is associated with increased frontal midline theta activity, correlating with attentional stability and reduced discursive thought (Cahn and Polich, 2006).

Vipassana, or “open monitoring” meditation, differs from Samatha. Vipassana meditators observe the uninterrupted flow of stimuli—sensations, thoughts and emotions—without labeling or reacting. Empirical findings indicate that people who have practiced Vipassana meditation for a long time show increased gamma brain activity in a specific brain area (parieto-occipital). This may relate to the adoption of a mindful and receptive awareness and enhanced perceptual clarity of moment-to-moment experience (Cahn et al., 2010). Additionally, mindfulness induces a sense of selflessness. Simultaneously, it reduces gamma activity in the prefrontal cortex, which serves as evidence suggesting that Vipassana meditation undermines the narrative ego (Dor-Ziderman et al., 2013).

Metta, or “loving-kindness” meditation, centers on cultivating unconditional goodwill and compassion toward oneself and others. Metta helps reshape the way we organize and experience our emotions. It can thereby reduce ingrained reactions such as hostility, anxiety, or fear (Hofmann et al., 2015; Stefan and Hofmann, 2019; Frick et al., 2020; Stangier et al., 2021). Metta meditation reduces self-other P300 amplitude differences in EEG/ERP studies, indicating balanced emotional processing. Moreover, long-term adherence to this practice engenders self-transcendence and concomitantly enhances interpersonal connectedness and altruistic tendencies (Trautwein et al., 2016).

Table 1 shows the different psychological mechanisms, neurophysiological correlates, and clinical outcomes for these three main meditation techniques. A key marker of this consciousness deepening is the shift from the “narrative self,” characterized by autobiographical identity and persistent internal dialogue, to the “minimal self,” which centers on momentary experience (Berkovich-Ohana et al., 2013, 2024). As practitioners move along this continuum, many report reduced cognitive fusion with personal stories and heightened awareness of immediate phenomena (Yang et al., 2024). Closely related to this transition is the cultivation of equanimity. Equanimity is a calm, balanced mind that stays steady no matter what thoughts or situations arise. Researchers measure equanimity as a key sign of advanced meditation, and they find that cultivating this quality can really change the way people deal with difficult situations (Desbordes et al., 2015; Hadash et al., 2016; Yang et al., 2024).

**TABLE 1:** Core Buddhist meditation techniques: mechanisms and outcomes.

Technique	Primary objective	Mechanism	Neural correlates	Clinical applications
Samatha	Attentional stability, absorption	Enhanced attentional control, reduced distractibility	↑Frontal midline theta (attentional control) (Cahn and Polich, 2006)	ADHD, attentional deficits (Neri et al., 2024)
Vipassana	Insight into impermanence, non-self *(anattā)*	Non-reactive observation of sensory/menta1 phenomena	↑Parieto-occipital gamma (Cahn et al., 2010); ↓Prefrontal gamma (Dor-Ziderman et al., 2013)	Depression treatment, chronic pain (Zeidan et al., 2019)
Metta	Cultivation of unconditional compassion	Affective training toward self/others	↓Self-other P300 amplitude difference (Trautwein et al., 2016);	Chronic depression (Stangier et al., 2021)

### 2.2 Psychological mechanism framework

Buddhist meditation’s effects can be illuminated by several psychological theories that detail its underlying mechanisms. These theories show that structured mindfulness practice can improve attention, influence emotions, and change how people see themselves. Thus, meditation builds a strong base for deeper self-discovery and personal growth.

At a foundational level, Meditation operates through dual cognitive systems: a reactive “hot” system driving impulsive responses during stress, and an adaptive “cool” system enabling reflective insights and flexible coping (Lehman et al., 2025). Meditation, specifically Samatha and Metta, appears to modulate this system through distinct pathways: the former enhances attentional stability and meta-awareness to reduce experiential fusion, while the latter cultivates prosocial affect and cognitive reappraisal to reinforce adaptive emotional patterns (Yordanova et al., 2020).

A growing body of research further suggests that what we commonly regard as a fixed “self” is better understood as a dynamic construct that can be broken down and reassembled over time (Berkovich-Ohana et al., 2024). Through consistent contemplative practice, the self-model can reorganize in non-linear ways, occasionally giving rise to experiences of “no-self” (anattā), where the usual boundaries of identity are perceived as porous or illusory (Laukkonen and Slagter, 2021; Cooper et al., 2022). Through its open-monitoring style, Vipassana meditation proves especially effective in cultivating the crucial insight. It brings to light the transient and constructed nature of thoughts, emotions, and bodily sensations (Dahl et al., 2015).

Finally, emerging frameworks in cognitive science, such as predictive coding, shed light on how meditation might recalibrate the brain’s Bayesian inferences (Lutz et al., 2019; Dutta, 2025). From this viewpoint, the mind perpetually generates predictions about incoming sensory information and updates them based on the precision of actual sensory data. Meditation practices, by reducing habitual patterns of reactive thought and enhancing mindful observation, can optimize this Bayesian inference process. As a result, practitioners become better at telling the difference between top-down expectations (our biases and beliefs) and bottom-up sensory signals (the raw data of experience). This refined capacity for sensory discrimination and error minimization contributes to a more vivid, less assumption-laden experience of reality (Manjaly and Iglesias, 2020).

Taken together, these theoretical models converge on a central theme: Buddhist meditation practices systematically reshape how individuals process and interpret their inner and outer worlds. From moderating emotional reactivity (dual-system theory) to reconfiguring the sense of self (PTS), or refining top-down and bottom-up perceptual loops (predictive coding), each framework underscores the transformative potential of contemplative training.

## 3 Empirical evidence: from neural correlates to clinical outcomes

Researchers are paying more attention to Buddhist meditation’s impact on the brain. Neuroimaging studies show advanced practitioners display distinctive brain activation and connectivity, especially in self-referential, attentional, and emotion-regulation networks (Guendelman et al., 2017; Guidotti et al., 2023). This leads to potential clinical benefits. Preliminary research suggests that practitioners who engage in long-term meditation experience less anxiety, depression, and stress. This section examines the evidence base, linking Buddhist contemplative methods to measurable outcomes in neuroplasticity, cognition, and therapeutic efficacy.

### 3.1 Neuroplasticity evidence

Extensive research indicates that Buddhist meditation promotes a flexible restructuring of the brain’s neural pathways. This process can lead to more stable attention, better emotional control, and enhanced meta-awareness. One of the most widely discussed findings involves modifications in the default mode network (DMN), a set of brain regions associated with self-referential thought and mind-wandering (Bremer et al., 2022). Through consistent mindfulness and concentrative practices, meditators enhance the ability to regulate interactions between the brain’s default mode network (DMN) and the salience network. This improves flexible shifts between internal reflection and external focus by optimizing attention allocation to relevant events (Raffone et al., 2019).

Additionally, long-term practitioners of Buddhist meditation exhibit strengthened connections in the brain circuits linking the prefrontal and parietal lobes (Kemmer et al., 2015; Yordanova et al., 2021). Meditation training consistently enhances theta synchronization in left parietal regions across practices (FAM/OMM/LKM), while beta-band connectivity in medial frontal networks distinguishes focused attention (FAM) from open monitoring (OMM). This reflects functional integration of fronto-parietal and cognitive monitoring networks, demonstrating meditation’s role in higher-order cognitive engagement beyond relaxation.

Electroencephalogram (EEG) and event-related potential (ERP) studies provide additional support for these findings by identifying neural signatures associated with specific meditation practices. Concentrative meditation, emphasizing sustained attention, demonstrates robust frontal midline theta enhancement, particularly in advanced practitioners. This theta activity, generated by anterior cingulate and prefrontal regions, indexes focused internal attention and reduced distractibility. While alpha increases may occur during relaxation, theta is the primary electrophysiological marker of concentrative proficiency, distinct from generalized alpha patterns associated with calm alertness (Cahn and Polich, 2006). Moreover, practitioners with extensive mindfulness retreat training demonstrate enhanced exogenous alerting in behavioral tasks, reflected by faster detection of uncued targets and reduced reliance on temporal warnings (Jha et al., 2007). These findings align with the idea that meditation training not only enhances focus but also modulates attention allocation by changing the neural dynamics of the brain (Lutz et al., 2004).

In the domain of higher meditative states, researchers observe a reduction in gamma-band power, an oscillation usually tied to demanding cognitive work (Dor-Ziderman et al., 2013). Its suppression in advanced meditation might reflect a refined meta-cognitive insight, wherein the practitioner observes mental content without becoming entangled or over-identified with it. This attenuation of gamma band activity could thus serve as an indicator of deeper introspective access and altered self-referential processing, linking to Buddhist concepts of selflessness or non-attachment.

### 3.2 Cognitive and behavioral effects

Beyond the neural level, a range of empirical studies has explored how such meditative training translates into cognitive and behavioral outcomes. Key among these is the enhancement of metacognition, or the capacity to monitor and regulate one’s own cognitive processes. Experienced meditators, for instance, detect the very first stirrings of intention with greater clarity (Jo et al., 2015; Lush et al., 2016). This suggests that Buddhist meditation might systematically cultivate introspective acuity, enabling practitioners to notice subtle mental cues before they escalate into broader courses of action.

In a similar way, meditation has been linked to a noticeable decrease in mind-wandering, which is the tendency for attention to stray from its main focus (Feruglio et al., 2021). By training attention in Samatha practice, individuals learn to sustain focus on a singular object or sensation (e.g., the breath) and to gently redirect stray thoughts back to the task at hand. Over time, this disciplined practice of returning to a focal point appears to restructure cognitive habits, resulting in less spontaneous wandering and a more deliberate mode of mental engagement.

The clinical implications of these cognitive changes are now well documented. In the realm of depression treatment, there is growing interest in combining Metta with mindfulness-based approaches (Frick et al., 2020). Metta, which cultivates loving-kindness and benevolence toward oneself and others, directly targets chronic depression by enhancing prosocial motivation and reducing self-criticism (Stangier et al., 2021). Pain management research also underscores the efficacy of Buddhist meditation. Functional MRI reveals that mindfulness deactivates thalamic pathways and default mode networks, reducing pain unpleasantness more effectively than hypnotic suggestions in some contexts (Zeidan et al., 2019; Wipplinger et al., 2023). Further studies are needed to compare specific meditation types (e.g., Vipassana vs. Metta) for targeted pain conditions and clarify their neural substrates.

## 4 Bridging paradigms: contrasting Buddhist and western psychological practices

Buddhist meditation and Western psychological approaches share goals like reducing distress and enhancing well-being. Yet, they differ in views of the mind, self-transformation, and ethical or philosophical matters. Comparing them reveals that each Buddhist pathways highlights ethics and deep introspection. This section contrasts hypnosis-induced attentional states and mainstream mindfulness interventions with Buddhist methods, highlighting differences in mental training. These contrasting perspectives ultimately complement and challenge each other in achieving holistic well-being. Table 2 lays out the major distinctions between Buddhist meditation, Western mindfulness, and hypnosis.

**TABLE 2:** Comparative analysis of contemplative practices.

Dimension	Buddhist meditation	Western mindfulness (MBIs)	Hypnosis
Philosophical basis	Integrated ethics *(sīla)*, non-self *(anattā)*, compassion	Secularized, detached from religious frameworks	Suggestion-based, no inherent ethical system
Primary goal	Consciousness transformation, ultimate liberation	Stress reduction, symptom management	Behavioral modification, symptom relief
Cognitive mechanism	Internally-driven meta-awareness	Present-moment awareness	Externally-induced “cold control” (Dienes, 2012)
Cultural adaptation	Context-dependent (e.g., monastic guidance)	Cross-cultural simplification; risk of “McMindfuhiess” (Kelly, 2023)	Culturally neutral
Key limitations	Advanced states (e.g., *nirodha samāpatti)* hard to quantify	Ethical dimensions overlooked; limited therapeutic depth	Dependent on suggestibility

### 4.1 Cognitive mechanisms compared to hypnosis

Buddhist meditation and hypnosis share surface-level similarities in eliciting absorption states and promoting autonomic regulation, often manifesting as relaxation that soothes body and mind. These parallels stem from both practices’ capacity to heighten focused attention or leverage suggestibility to guide experiential states (Holroyd, 2003; Penazzi and De Pisapia, 2022). In hypnosis, externally driven suggestions engage cognitive pathways aligned with “cold control” theory (Dienes, 2012; Lush and Dienes, 2019). Under this model, these suggestions temporarily disconnect executive control from conscious awareness, such that people feel their actions occur automatically.

Conversely, Buddhist meditation employs internally guided techniques (e.g., Samatha or Vipassana) to cultivate meta-awareness, allowing practitioners to observe thoughts, emotions, and sensations non-judgmentally (Lehman et al., 2025). Although both practices can lead to altered states, the underlying metacognition is opposite. Hypnosis involves inaccurate metacognition of intentions (experiencing voluntary actions as involuntary), while meditation cultivates enhanced metacognitive access to intentions. Thus, hypnosis relies on disrupted higher-order awareness of volition, whereas meditation refines conscious insight into intentional processes (Lush and Dienes, 2019).

### 4.2 Distinctions from mainstream mindfulness interventions

Modern mindfulness-based interventions (MBIs) are usually presented as secular tools for stress relief and symptom control. This contrasts with traditional Buddhist mindfulness, which is deeply intertwined with moral and ethical precepts in texts such as the Vinaya and the Abhidhamma (Shulman, 2024; Rohde et al., 2024). In Buddhist contexts, ethical conduct, compassion, and community life are foundational elements that shape how mindfulness is cultivated and applied. When these aspects are removed for secular therapeutic settings, the practice may lose some of its transformative potential and philosophical depth.

Furthermore, second-generation mindfulness-based interventions (SG-MBIs) seek to integrate contemplative elements of Buddhist psychology—such as loving-kindness and non-self teachings—into clinical protocols (Van Gordon and Shonin, 2020; Gamaiunova et al., 2022). These SG-MBIs strive to go beyond mere symptom alleviation by exploring deeper existential or spiritual dimensions. In doing so, they underscore the importance of a holistic and ethically grounded framework, thus representing a closer parallel to the original intentions and scope of Buddhist meditation while still addressing contemporary clinical needs.

## 5 Critical gaps and future directions

Despite a large number of studies on Buddhist meditation emerging in recent years, which have greatly enhanced our knowledge of this ancient practice, progress is still impeded by a stubborn gap between subjective reports (e.g., emergent phenomenology) and objective measurements (e.g., neurobiological states). A central challenge is bridging contemplative frameworks—such as the Buddhist doctrine of not-self (anattā)—with empirical paradigms to develop testable models of mental phenomena (Wright et al., 2023). Reliance on self-report data risks measurement bias and incomplete insights, highlighting the necessity of complementary behavioral methodologies (Nyklíček, 2020). Such approaches reveal divergent aspects of self-awareness—undetectable via questionnaires—and demonstrate added value in capturing nuanced phenomenological experiences like interoception during spontaneous states.

Addressing these gaps requires practical strategies that emphasize cultural sensitivity. For example, the concept of “mindfulness” varies significantly between traditions. Many Buddhist schools associate sati with ethics and philosophy, whereas Western psychology treats it mainly as an attentional or cognitive-behavioral skill (Krägeloh, 2018; Cassaniti, 2022). When there is disagreement among people regarding the intended objectives of meditation, it leads to ambiguity about its purpose. Consequently, this ambiguity permeates the planning of studies and the application of clinical treatments. Additionally, Western-developed scales may fail to capture the ethical or relational dimensions essential to practices like Metta or Chinese Chan (Zen) meditation. Hirayama et al. (2014), for instance, found that direct translations of the Freiburg Mindfulness Inventory were insufficient to encompass essential aspects of Eastern contemplative experiences. Equally crucial is guarding against the commodification of mindfulness, which Kelly (2023) argues risks reducing meditation to a superficial, market-driven commodity (“McMindfulness”). This commercialization not only dilutes mindfulness’s transformative potential but also fosters “spiritual bypassing.”

Interdisciplinary collaborations promise new insights into how meditation shifts consciousness at both subjective and neural levels. Methodological innovations from neurophenomenology offer a way to synchronize first-person reports of meditative experience with objective neurobiological measures (Lutz et al., 2024; Yang et al., 2025). In parallel, Bayesian predictive coding models demonstrate that Insight meditation enhances the brain’s capacity for self-assessment. This improvement is likely to facilitate the reversal of detrimental learning patterns in individuals with functional neurological disorders (Dutta, 2025). Bringing predictive coding together with meditation research will allow future studies to map in detail how Buddhist contemplative methods transform cognition, emotion, and self-perception.

Overall, investigations into Buddhist meditation have consistently reported robust effects on neural networks, emotional regulation, and meta-cognitive awareness. However, cultural and research-related challenges still persist, which means we require more advanced measurement tools and analytical methods. Strengthening interdisciplinary ties, embracing culturally informed approaches, and upholding ethical rigor are essential to unlocking the full potential of Buddhist contemplative methods in both clinical and broader societal contexts.

## 6 Conclusion

In conclusion, this review underscores how Buddhist meditation offers a transformative pathway that extends beyond conventional mindfulness. By systematically cultivating concentrative focus (Samatha), insight-oriented awareness (Vipassana), and compassion-based practices (Metta), practitioners can progressively deepen consciousness and develop refined meta-cognitive insights. The integration of attention training, emotional regulation, and self-inquiry emerges as a distinct framework that can help dissolve habitual thought patterns and foster greater authenticity in daily life. Notably, these practices point toward insights like no-self (anattā), challenging the limitations of self-report measures that may fail to capture the breadth and depth of such advanced experiences.

To strengthen validation of subjective accounts of transformative states, researchers and practitioners ought to collaborate on the design and application of objective or semi-objective methodologies. Such approaches may encompass carefully controlled behavioral tasks as well as precise introspective timing paradigms. In doing so, they can support practitioners’ observations and minimize possible biases in collecting data. Future research will benefit from culturally sensitive and ethically informed approaches, ensuring diverse populations are included and respected. Ultimately, bridging traditional Buddhist contemplative insights with modern scientific investigations may yield a more robust understanding of consciousness, informing therapeutic interventions and enhancing mental health across various contexts.
